# Carbonic anhydrase IX in tumor tissue and sera of patients with primary cervical cancer

**DOI:** 10.1186/1471-2407-11-12

**Published:** 2011-01-11

**Authors:** Linn Woelber, Kerstin Kress, Jan F Kersten, Matthias Choschzick, Ergin Kilic, Uwe Herwig, Christoph Lindner, Joerg Schwarz, Fritz Jaenicke, Sven Mahner, Karin Milde-Langosch, Volkmar Mueller, Maike Ihnen

**Affiliations:** 1 Department of Gynecology and Gynecologic Oncology, University Medical Centre Hamburg-Eppendorf, Martinistrasse 52, 20246 Hamburg, Germany; 2 Department of Medical Biometry and Epidemiology, University Medical Centre Hamburg-Eppendorf, Martinistrasse 52, 20246 Hamburg, Germany; 3 Institute of Pathology, University Medical Centre Hamburg-Eppendorf, Martinistrasse 52, 20246 Hamburg, Germany; 4 Department of Gynecology, Albertinen Hospital, Süntelstrasse 11A, 22457 Hamburg, Germany; 5 Department of Gynecology, DKH Hospital, Hamburg, Hohe Weide 17, 20259 Hamburg, Germany; 6 Department of Gynecology, Asklepios Clinic Nord, Tangstedter Landstraße 400, 22417 Hamburg, Germany

## Abstract

**Background:**

Carbonic anhydrase IX (CAIX) is a membranous expressed metalloenzyme involved in pH homeostasis and cell adhesion. The protein is overexpressed in a variety of tumors and potentially associated with negative outcome. This study was designed to investigate the prognostic role of CAIX in serum and tumor tissue of patients with primary cervical cancer.

**Methods:**

Tumor samples of 221 consecutive patients with primary cervical cancer who underwent surgery between 1993 and 2008 were analyzed for CAIX expression by immunohistochemistry. Additionally, preoperative serum CAIX concentrations were determined by ELISA in a subset of patients. Correlation with intratumoral CAIX expression as well as clinicopathological factors and outcome was analyzed.

**Results:**

CAIX expression was observed in 81.9% of the tumor specimens; 62.0% showed a moderate or strong staining intensity. Moderate/strong expression was associated with squamous histology (p = 0.024), advanced tumor stage (p = 0.001), greater invasion depth (p = 0.025), undifferentiated tumor grade (p < 0.001) and high preoperative SCC-Ag values (p = 0.042). Furthermore patients with moderate/strong intratumoral CAIX expression had a higher number of metastatic lymph nodes compared to those with none/weak intratumoral expression levels (p = 0.047) and there was a non-significant association between high intratumoral CAIX expression and shorter survival (p = 0.118). Preoperative serum concentrations of CAIX ranged between 23 and 499 pg/mL and did not correlate with intratumoral expression or other clinicopathological variables.

**Conclusion:**

CAIX is associated with advanced tumor stages and lymph node metastases in cervical cancer, potentially representing a new target in this disease. In contrast to other epithelial cancers we could not observe a correlation between serum CAIX and its intratumoral expression.

## Background

Altered glycolysis is one of the crucial characteristics of cancer cells [1]. While normal cells can suppress the conversion of pyruvate to lactic acid in the presence of oxygen, cancer cells bypass oxidative phosphorylation in mitochondria for ATP production. The intracellular lactate excess results in acidification of the extracellular space and upregulation of acid-controlling proteins like carbonic anhydrases [2]. They catalyze the hydration of CO_2 _to HCO_3 _and play a central role in pH homeostasis [3,4]. Carbonic anhydrase IX (CAIX) is one of the members of this enzyme family; apart from its role in the pH regulation, CAIX has a function in cell adhesion and is important for growth and survival of tumor cells [5-7]. CAIX expression is induced by hypoxia through stabilization of the transcription factor HIF-1α [8]. In normal tissue, expression of CAIX is very limited, low levels have been detected in gastric cells, the epithelium of the gall bladder, pancreatic ducts or in crypt cells of the small intestine [9]. Despite its sparse expression in normal tissue, CAIX is overexpressed in a variety of solid tumors [10]. High levels of CAIX were found to be associated with unfavourable patient outcome in several epithelial cancers [11-14]. In renal cell cancer, overexpression of CAIX is common and the possible role of CAIX targeting antibodies (WX-G250, Rencarex^®^) is currently evaluated in phase III trials [15].

Besides the cell-associated membranous form of CAIX, there is a soluble isoform that is released by proteolytic cleavage and can be detected in the serum [16]. This form of CAIX could serve as an easily accessible marker to stratify patients for therapy and monitor response. Information regarding the soluble form of CAIX is very limited [17]. There are studies on serum CAIX in renal cell cancer patients showing significantly higher concentrations in patients with metastatic disease than in patients with localized cancer [16,18]. Furthermore, renal cell cancer patients with high serum CAIX before surgery appear to be at significantly higher risk for disease recurrence than those with low preoperative values [18]. Other authors could show that CAIX was cleared from serum after complete tumor resection, suggesting a very promising marker to monitor therapy response and disease recurrence [16]. In vulvar cancer we could demonstrate similar results: Preoperative serum CAIX levels correlated with intratumoral CAIX expression and high values were prognostic for unfavourable outcome [19].

Cervical cancer is the third most common cause of cancer-related death in women accounting for approximately 500,000 new cases and 280,000 deaths worldwide each year [20]. Currently established prognostic factors include International Federation of Gynecology and Obstetrics (FIGO) stage, tumor volume, lymphatic spread, human papilloma virus (HPV) type 18 infection, invasion depths and vascular space invasion [21-23]. Although these prognostic factors are well established, the biological factors associated with recurrence and survival remain largely unknown and treatment options for recurrent or advanced disease are limited especially in patients already treated with radiochemotherapy. The identification of new prognostic factors and markers for therapy monitoring is therefore of high clinical relevance. In daily routine, serum Squamous Cell Cancer Antigen (SCC-Ag) is often used to monitor therapy of advanced disease [24]. Its value as a sensitive and specific tumor marker has been previously demonstrated [24-26]. The role of CAIX in cervical cancer has not been fully determined. Overexpression is potentially associated with a higher rate of lymph node metastases and with negative prognosis [27-29]. Serum CAIX could therefore be of clinical relevance for prognosis and therapy monitoring in cervical cancer. However, information on serum CAIX in cervical cancer is not available.

To gain insight into the role of intratumoral and serum CAIX in cervical cancer, we determined CAIX expression and its prognostic relevance in correlation with preoperative CAIX serum concentrations and other clinicopathological factors as well as survival in patients with primary cervical cancer.

## Methods

### Patients

Tumor tissue from 221 patients undergoing surgery for primary cervical cancer at 4 different institutions between 1993 and 2008 was analyzed. Detailed patient characteristics are listed in Table 1. In addition, 46 preoperative serum samples from a subset of the included patients were analyzed. Serum samples were not routinely taken from all included patients before surgery but on a voluntary basis in one of the participating centers. To assure the analysis of a representative collective, FIGO stages were compared to those of the whole cohort showing a similar stage distribution (FIGO stage Ib1 52.2%, Ib2 13.0%, IIa 6.5%, IIb 17.4%, III 0.0%, IVa 4.3%, IVb 6.5%, compare Table 1). Clinicopathologic factors were evaluated by reviewing medical charts and pathologic reports. For tumor staging, 6^th ^edition UICC TNM classification and stage groupings were used [30]. Informed consent for the scientific use of patient data, tumor tissue and serum had been obtained from all patients in coordination with the local ethics committee according to the principles of the declaration of Helsinki (Ethics committee of the Medical Board Hamburg, reference number #190504). In the current study, all data were analyzed anonymously.

**Table 1:** Patient characteristics (n = 221)

		(%)
*Age (years)*		
Median	47	
Mean	49	
Range	23 - 85	

*Histological subtype (n = 221)*		
Squamous cell carcinoma	161	(72.9)
Adenocarcinoma	35	(15.8)
Adenosquamous cell carcinoma	25	(11.3)

*Tumor stage (FIGO) (n = 221)*		
Ia1	0	(0.0)
Ia2	3	(1.4)
Ib1	113	(51.1)
Ib2	20	(9.0)
IIa	17	(7.7)
IIb	44	(19.9)
IIIa	0	(0.0)
IIIb	1	(0.5)
IVa	7	(3.2)
IVb	16	(7.2)

*Lymph node involvement (n = 221)*		
N0	168	(76.0)
N1	53	(24.0)

*Removed lymph nodes (n = 221)*		
Median	27	
Mean	30	
Range	2 - 88	

*Affected lymph nodes (n = 53)*		
Median	2	
Mean	3	
Range	1 - 14	

*Distant metastasis (n = 181)*		
M0	165	(91.2)
M1	16	(8.8)

*Tumor grade (n = 215)*		
G1	9	(4.2)
G2	95	(44.2)
G3	111	(51.6)

*Lymphovascular space invasion (n = 196)*		
L0	64	(32.7)
L1	132	(67.3)

*Vascular invasion (n = 169)*		
V0	151	(89.3)
V1	18	(10.7)

*Surgical therapy (n = 181)*		
Radical hysterectomy	180	(99.5)
Trachelectomy	1	(0.5)

*Preoperative SCC serum concentration (μg/l) (n = 144)*		
Median value	1.4	
Mean	5.2	
Range	0.2 - 107.0	

*Adjuvant Therapy (n = 180)*		
No therapy	88	(48.9)
Adjuvant radiotherapy	44	(24.4)
Adjuvant concomitant chemoradiotherapy	48	(26,7)

*Follow-up (months) (n = 175)*		
Median	49	
Mean	63	
Range	2-193	
*Disease free surviving*	131	(74.9)
*Disease recurrence*	44	(25.1)
*Died of disease*	38	(21.7)

*CAIX expression (IRS) (n = 221)*		
Median expression	2	
Mean	3	
Range	0 - 12	
IRS 0	40	18.1
IRS 1	44	19.9
IRS 2	35	15.8
IRS 3	9	4.1
IRS 4	32	14.5
IRS 6	29	13.1
IRS 8	2	0.9
IRS 9	23	10.4
IRS 12	7	3.2

*CAIX serum concentration (pg/mL) (n = 46)*		
Median concentration	104	
Mean	137	
Range	23-499	

IRS: Immunoreactive score (IRS) according to W. Remmele and H. E. Stegner

For 180 patients detailed therapy information and for 175 patients detailed follow-up data from the date of primary surgery to the date of death or last contact (July 2009) were available. The treatment of cervical cancer during the investigational period consisted of radical hysterectomy and resection of the pelvic and paraaortic lymph nodes via laparotomy for FIGO stages I and II. Only in stage Ia and Ib disease with fertility preserving objective radical hysterectomy was omitted. Adjuvant radio/chemotherapy of the pelvis and the paraaortic region was performed in case of advanced disease according to effective German Guidelines [31]. For FIGO stage III and IV cervical cancer primary radio/chemotherapy was generally recommended. In our study, 24 patients were included with FIGO stage III/IV disease who received primary surgery. Those patients underwent surgery either to prevent further tumor invasion of the bladder and/or rectum or were not classified as FIGO III/IV until surgery (predominantly in case of paraaortic lymph node metastases).

### CAIX Immunohistochemistry

To analyze the tissue-expression of CAIX, we performed immunohistochemistry (IHC) with paraffin embedded tumors from all included patients, using a commercially available CAIX IHC kit (Oncogene Science, Cambridge, Mass 0214, USA) according to the manufacturer's instructions. Tissue sections were cut at a thickness of 5 μm and mounted on slides (Superfrost/Plus, Sondheim Germany), dewaxed with Xylene and gradually hydrated. Afterwards a peroxidase blocking solution was applied and sections were incubated at room temperature for 5 minutes followed by several washings in phosphate buffered saline (PBS) (CA IX IHC Kit, Reagent H). Adjacent all sections were probed with the primary mouse anti-CA IX antibody (prediluted at 0.5 μg/mL, CA IX IHC Kit, Reagent B) incubating them at room temperature for 30 minutes. After several washings in PBS, slides were covered with the biotinylated secondary antibody (CA IX IHC Kit, Reagent C) for 10 minutes, washed several times and incubated with HRP-labeled streptavidin (CA IX IHC Kit, Reagent D) for another 10 minutes to be washed three times again. Finally the DAB substrate-chromogen solution (CA IX IHC Kit, Reagent F1, F2 and F3) was applied for 5 minutes followed by washings in Aqua dest. The slides were briefly counterstained with haematoxylin (CA IX IHC Kit, Reagent G) and dehydrated before mounting.

The IHC staining was evaluated independently by two gynecopathologists using the immunoreactive score (IRS) according to W. Remmele and H. E. Stegner as product of staining intensity (graded 0-3) and percentage of positive cells (graded 0-4, 0: 0%, 1: <10%, 2: 10-50%, 3: 51-80%, 4: >80%) resulting in a score from 0-12 [32].

### Quantitative analysis of serum CAIX level

CAIX was quantified by a commercially available ELISA (Oncogene Science, Cambridge, Mass 0214, USA). Control samples consistent of purified CAIX protein in buffer with low, medium and high concentrations as included in the ELISA kit ("MN/CAIX ELISA controls"). Serum samples and controls were diluted 1:2 with sample diluting buffer (containing bovine serum albumin, mouse IgG, buffer salts and 0.09% sodiumazide). One hundred microliters of the standards, of diluted control samples and of diluted serum samples were dispensed into the wells of a 96-well plate (coated with the monoclonal capture antibody) and incubated for two hours at room temperature on a shaker at 800 rpm. Wells were washed, and 100 μl of the detection antibody (containing biotinylated anti-CAIX antibody) was added. The plates were incubated for 30 min at room temperature, washed, and then further incubated with 100 μl of a streptavidin-HRP (horseradish peroxidase) conjugate for 30 min at room temperature. After washing, 100 μl of chromogenic substrate (TMB blue substrate) was added for 30 min at room temperature. The reaction was stopped with 100 μl of 2.5 N sulphuric acid and absorbance was read at 450 nm by an automated plate reader (Tecan, Crailsheim, Germany). The CAIX concentration was estimated from the standard curve. Each sample, standard, and control was analyzed in duplicate.

### Statistics

All statistical analyses were conducted using the R open source statistical software package [33]. p-values < 0.05 were considered statistically significant. In accordance with previously published data on CAIX expression in epithelial tumors we analyzed CAIX expression in a two class system, merging IRS score 0 and 1 into one class corresponding with none/weak CAIX expression levels and IRS score 2 - 12 into another class corresponding with moderate/strong intratumoral CAIX expression [19,34,35]. Mann-Whitney-U-Tests and Fisher's exact tests were used to calculate correlations between CAIX expression, serum CAIX and clinicopathological parameters. Kaplan-Meier analyses of overall and disease-free survival were performed. The log rank test was applied to examine the relationship between serum CAIX, intratumoral CAIX expression and survival. Additionally, we performed a stratified log rank test, comparing CAIX effects on survival in patients with FIGO stage I and FIGO stage II-IV disease, to investigate potential stage dependant differences. The Cox Proportional Hazard Model was used for multivariate analyses.

## Results

### Patients

A total of 221 patients were included in this study; detailed characteristics are listed in Table 1. For 180 patients detailed therapy information and for 175 patients detailed follow-up data were available. All patients underwent radical surgery. No further treatment was initiated in 88 patients (48.9%), whereas 92 (51.1%) patients received adjuvant radio- and/or chemotherapy. The predominantly applied chemotherapy regimen was cisplatin as a single agent, 4 patients received a cisplatin based combination therapy. Median follow-up time was 49 months. At the time of last follow-up, 131/175 (74.9%) patients were alive and without relapse whereas 38/175 (21.7%) patients had died from the disease. In case of disease recurrence (n = 44), relapse was locoregional in 10 patients, distant in 13 patients and both in 18 patients (3 cases unknown).

### CAIX immunohistochemistry

**Figure 1:**
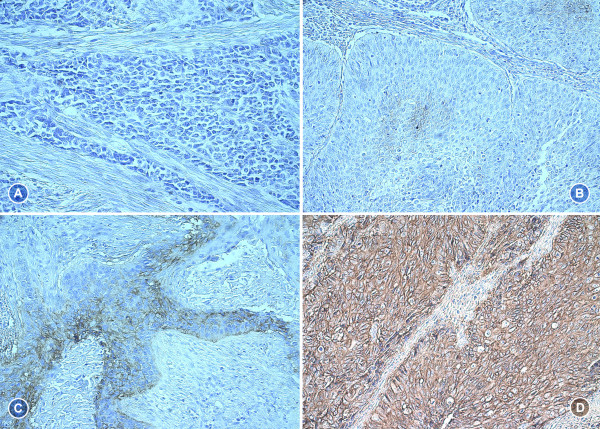
**CAIX IHC: none (A, IRS 0), weak (B, IRS 1), moderate (C, IRS 4) and strong (D, IRS 12) expression**.

**Figure 2:**
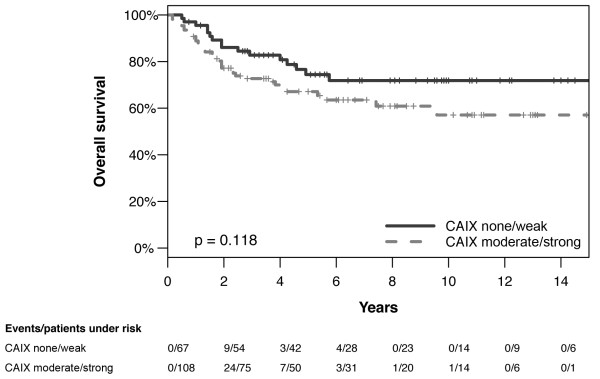
**Overall survival of patients with moderate/strong versus vs. none/weak intratumoral CAIX expression (p = 0.118, n = 175)**.

A total of 221 tumor samples were stained for determination of CAIX expression. CAIX was detectable in 181 (81.9%) of all specimens. 137 (62.0%) samples exhibited a moderate or strong (score 2-12) staining result (Table 1). Figure 1 shows representative tumor samples with none/weak/moderate/strong CAIX expression. CAIX protein expression was always membranous. Tumor associated stromal cells as well as inflammatory cells stained negative for CAIX. Table 2 summarizes the correlation of CAIX expression with clinicopathological parameters. Moderate/strong expression was associated with squamous histology (p = 0.024), advanced FIGO and pT stage (p = 0.013 and p = 0.001), greater invasion depth (p = 0.025), undifferentiated tumor grade (p < 0.001) and high preoperative SCC-Ag concentration (p = 0.042). Furthermore, patients with moderate/strong intratumoral CAIX expression had a higher number of metastatic lymph nodes compared to those with none/weak intratumoral expression levels (p = 0.047). Fifteen (11.0%) of the patients with moderate/strong intratumoral CAIX expression showed three or more lymph node metastases whereas in only 2 cases (2.4%) with none/weak CAIX expression three or more positive lymph nodes were diagnosed. We could not demonstrate an association between CAIX expression and lymphovascular space invasion or patients' age. There was an association between none/weak intratumoral CAIX expression and longer survival; however, this result failed to reach statistical significance (overall survival p = 0.118, Figure 2; disease-free survival p = 0.220, not shown). The potential negative effect of a moderate/strong intratumoral CAIX expression on survival did not significantly differ between FIGO stages I and II-IV in a stratified survival analysis (disease-free survival p = 0.580, overall survival p = 0.331, not shown).

**Table 2:** Correlations between CAIX expression and clinicopathological factors.

		Carbonic anhydrase IX expression		p-value
		**none/weak**	**moderate/strong**	

**All samples (n = 221)**		84	137	

*Histological subtype *(n = 221)	squamous	53	108	**0.024**
		
	adenomatous	20	15	
		
	adenosquamous	11	14	

*FIGO stage *(n = 221)	I	62	74	**0.013**
		
	II	15	46	
		
	III/IV	7	17	

*UICC tumor stage *(n = 221)	pT1	65	76	**0.001**
		
	pT2	15	55	
		
	pT3/4	4	6	

*Nodal involvement *(n = 221)	pN0	69	99	0.106
		
	pN1	15	38	

*Number of positive lymphnodes *(n = 221)	0	69	99	**0.047**
		
	1-3	13	23	
		
	>3	2	15	

*Tumor grade *(n = 215)	G1	9	0	**<0.001**
		
	G2	32	63	
		
	G3	39	72	

*Lymphovascular space invasion *(n = 196)	L0	25	39	0.875
		
	L1	49	83	

*Invasion depth in mm *(n = 185)*	median	12 (n = 70)	16 (n = 115)	**0.025**

*Patients age in years *(n = 221)*	median	49.5	47.0	0.549

*SCC-Ag in μg/L *(n = 144)*	median	1 (n = 55)	1.9 (n = 89)	**0.042**

*Serum CAIX in pg/mL *(n = 46)*	median	91 (n = 17)	110 (n = 29)	0.466

Fisher's exact test was used for categorical data, the Mann-Whitney-U-Test for continuous variables (*).

### Serum CAIX

**Figure 3:**
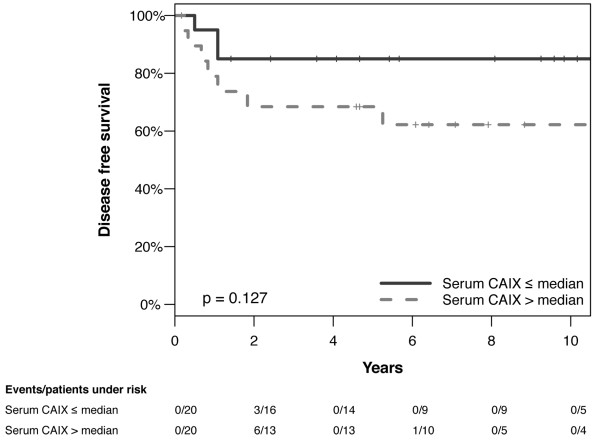
**Disease-free survival of patients with Serum CAIX below and above the median of 104 pg/ml (p = 0.127, n = 40)**.

**Figure 4:**
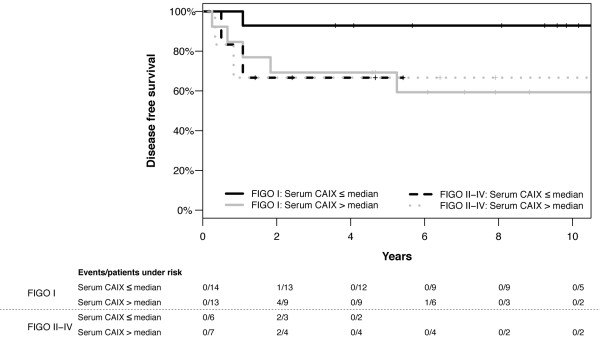
**Disease-free survival of patients with Serum CAIX below and above the median of 104 pg/ml stratified according to FIGO stage (p = 0.051 and p = 0.919, n = 40)**.

Median preoperative serum CAIX was 104 pg/mL (mean 137.0, range 23-499 pg/mL). Patients with a moderate/strong intratumoral CAIX expression did not show significantly higher serum concentrations (median 110 pg/mL, range 23-499 pg/mL) compared to patients with none/weak expression (median 91 pg/mL, range 27-257 pg/mL, p = 0.466). Furthermore, serum CAIX did not show any association with the analyzed clinicopathological factors (tumor stage, lymph node involvement, lymphovascular space invasion, invasion depth, tumor grade, histological type, preoperative serum SCC-Ag). Nevertheless, there was a non-significant association between high preoperative serum CAIX and shorter disease-free survival (disease-free survival p = 0.127, Figure 3; overall survival p = 0.320, not shown). After stratifying for FIGO stages, the negative effect of a serum CAIX above median on survival was especially distinct in patients with FIGO stage I disease (disease-free survival p = 0.051, Figure 4; overall survival p = 0.059, not shown)

### Multivariate analysis

The independent prognostic impact of intratumoral CAIX expression and preoperative serum CAIX for disease-free and overall survival adjusted for tumor stage (FIGO), pN, tumor grade and depth of invasion was analyzed using the Cox Proportional Hazard Model. Higher FIGO stage and nodal involvement were shown to be independent prognostic factors for decreased survival. Neither moderate/strong CAIX expression nor serum CAIX above median before surgery could be demonstrated independently relevant for prognosis in this patient cohort.

## Discussion

In this study we analyzed the prognostic relevance of CAIX in tumor tissue of 221 and sera of 46 patients with primary cervical cancer, representing the largest study on CAIX expression in cervical cancer and the first study investigating the role of serum CAIX for this tumor entity. The high number of samples in combination with survival data enables a comprehensive survey of CAIX expression with respect to multiple clinicopathological parameters.

More than 80% of the patient samples stained positive for CAIX (62% with moderate/strong intensity). This expression rate confirms previously published results: In a study by Loncaster et al, expression was found in 79% of the samples [13,36]. Tumor associated stromal cells as well as inflammatory cells stained negative for CAIX. In our analysis, moderate/strong intratumoral CAIX expression was significantly associated with advanced tumor stages, a higher depth of invasion, undifferentiated tumor grade as well as higher preoperative serum SCC levels. Furthermore, there was a strong correlation of CAIX expression to the number of positive locoregional lymph nodes. All of these features are well known to be associated with unfavorable clinical outcome [21,22]. These results are confirmed by published results from smaller studies on CAIX expression in cervical cancer [27-29]. Up-regulation of CAIX is probably an adaptation to aerobic glycolysis in tumor cells for maintenance of the intracellular pH in advanced carcinomas. Acidification of extracellular space by this mechanism may contribute to tumor cell invasion and development of metastases [37,38]. High levels of CAIX may therefore promote the development of locoregional lymph node metastases. In our study we also found an association between moderate/strong intratumoral CAIX expression and shorter survival. The effect was not significant, possibly because of a restricted number of patients with advanced tumor stages and/or positive lymph nodes. Taken together, enhanced expression of CAIX appears to be an important feature of cancerogenesis in cervical cancer.

Serum CAIX could therefore serve as an easily accessible marker to stratify patients for therapy and monitor response. While tumor tissue is usually only available at surgery, serum is easily accessible during the whole course of the disease. This is especially relevant in patients that are not always treated with a primarily surgical approach, as for example in advanced cervical cancer. Currently, detailed information on serum CAIX as a biomarker is only available in renal cell cancer patients: Serum CAIX has been shown to serve as a promising marker for therapy monitoring and prediction of prognosis in patients with cancer of the kindney [16]. The marker is especially interesting as some groups have reported a clearance of CAIX from serum after complete tumor resection [16]. However, in a longitudinal study on serum CAIX in ovarian cancer patients at our institution, we could not observe a clearance of CAIX from serum after surgery and chemotherapy [17].

To investigate the role of serum CAIX in cervical cancer we determined preoperative serum concentrations in a subset of 46 patients. Patients in our study showed CAIX serum concentrations between 23 and 499 pg/mL with a mean of 137 pg/mL. Patients with renal cell carcinoma had similar concentrations in localized disease though concentrations were measured using a different assay (91.65 ± 13.29 pg/mL) [18]. In our own series on preoperative serum CAIX in vulvar cancer analyzed by the same assay, serum concentrations (range 56 and 879 pg/mL, mean 237.29 pg/mL) were above those found in the current study despite mostly locally restricted disease [19]. A defined cut-off level for normal values of serum CAIX has not been determined yet and conflicting results have been published concerning CAIX serum levels in healthy males and females. In the series of Li et al. mean serum CAIX level was significantly higher in renal cell cancer patients than in healthy individuals (patients with metastatic renal cell cancer: mean 216.68 ± 67.02 pg/mL; patients with localized disease: mean 91.65 ± 13.29 pg/mL and healthy individuals: mean 14.59 ± 6.22 pg/mL). In contrast to these findings, others reported considerably higher levels in normal sera (mean 221 pg/mL in healthy females) [39].

Information regarding the relation of intratumoral CAIX expression and detection of its soluble isoform is very limited. A recently published study on CAIX expression and serum concentrations in renal cell cancer could not demonstrate a correlation: Zhou et al. found serum CAIX to be associated with tumor size but not with intratumoral CAIX expression [40]. Possibly, intratumoral hypoxia, increasing with tumor size, causes the soluble form of CAIX to be released into bloodstream. In contrast to these results, a study on urinary CAIX in renal cell cancer patients could demonstrate a coherence of soluble CAIX and intratumoral CAIX expression [41]. Both studies are based on small patient numbers. In our previous study on vulvar cancer, patients with high serum CAIX showed a significantly higher intratumoral expression of CAIX while the current study fails to proof this correlation for cervical cancer. One possible explanation could be the low proportion of patients with advanced disease.

The prognostic role of the soluble form of CAIX in cancer patients is extremely limited. We observed a non-significant association between high CAIX serum concentrations before surgery and unfavourable disease-free survival; this effect was especially seen in early stage disease. Our findings are consistent with results on CAIX serum concentrations in renal cell cancer: Li et al. could demonstrate a decreased disease-free survival for patients with high serum CAIX concentrations before surgery [18]. However, in our longitudinal study on serum CAIX in ovarian cancer patients, we could not observe a prognostic relevance of preoperative serum CAIX for survival [17].

## Conclusions

CAIX overexpression is seen in a great proportion of patients with cervical carcinoma and related to adverse clinicopathological parameters as well as unfavorable outcome. The exclusive expression of CAIX in cancer tissue qualifies this protein as suitable target for therapeutic interventions. The role of serum CAIX in this context remains uncertain; a detailed evaluation of this serum marker in greater cohorts is highly desirable to clarify its clinical role.

## Competing interests

Volkmar Mueller has received research support by supply of ELISA systems from Oncogene Science at no cost. The other authors declare that they have no competing interests involved with the presented data. This study was funded by internal departmental sources. CAIX immunohistochemistry and ELISA kits were supplied by Oncogene Science without restrictions and participation in study design or data analyses.

## Authors' contributions

All authors have made substantive intellectual contributions to the study and given final approval of the final manuscript to be published.

LW has designed the study, statistically analyzed and interpreted the data and drafted the manuscript. KK, KM and MI were responsible for data acquisition, carried out the immunoassays and helped to draft the manuscript. MC and EK carried out the IHC and participated in the design and coordination of the study. JFK carried out statistical analyses, participated in the design of the study and helped to draft the manuscript. UH, CL and JS were involved in data acquisition and revised the manuscript critically. FJ and SM participated in the design and coordination of the study and the interpretation of the results. VM helped to draft the manuscript, participated in the design and coordination of the study and the interpretation of the results.

## Pre-publication history

The pre-publication history for this paper can be accessed here:

http://www.biomedcentral.com/1471-2407/11/12/prepub
